# British Lunatic Asylums

**Published:** 1851-10-01

**Authors:** 


					Art. IV.?
BRITISH LUNATIC ASYLUMS.
We have before us the Annual Reports of the principal lunatic asylums
of Great Britain. They constitute, apart from the parliamentary returns
of the Commissioners in Lunacy, our only accessible record of the
actual condition of the large body of insane persons confined in the
public or county asylums of this country. The Reports, speaking of
them collectively, are drawn up with ability, and with great attention
to statistical accuracy. Some of the documents are meagre?many of
them, however, are full and elaborate, but all deserve praise for the
kindly spirit in which they are written. It has been alleged by those
ever ready to censure and condemn, that the Reports of the English
asylums have only one redeeming quality?viz., their brevity. It
should, however, be borne in mind, that if this be the fact, the medical
officers are not always to be blamed for the paucity of information
communicated in their official returns. In some instances the com-
mittee of management positively refuse to have any Report at all pub-
lished, and, in other cases, they restrict the medical superintendent as
to the length of their Reports. It is unnecessary for us to exclaim
against this questionable exercise of the spirit of economy. Our
national asylums, supported by the public purse, should constitute our
great schools of mental pathology. How is this to be effected if the
medical officers are inefficiently supported by those appointed to carry
out the general objects of these important charities? To begrudge the
trifling expense of printing an annual Report of the condition of a
public lunatic asylum appears almost incredible, and yet such we are
assured is the fact. We trust it will not be our duty to revert to this
subject again. The medical staff of some of our public asylums is not
only notoriously defective in strength, but is overworked and in some
instances miserably underpaid.
Having made these few prefatory remarks, we proceed to analyze the
LEICESTER LUNATIC ASYLUM. ' 537
documents before us. The medical Report of the Leicestershire and
Rutland Asylum communicates the following statistical data:?
Patients on the Books, December 31,1819 200
Admitted since 101
307
Discharged?Cured 49
Relieved 13
62
26
Died?Epilepsy   2
General Paralysis  9
Age and Debility  2
Maniacal Exhaustion  (i
Other Causes  7
Remaining?County Paupers 163
Out County Paupers  8
Subscribers ......... 33
Independent 15
219
307
Total number admitted since the opening of the Institution. 1139
Total number Cured,   505
Relieved .   98
Removed 105
Died 212
Remaining 219
1139
It appears that a reduction in the weekly rate of maintenance has
taken place. " The rate of maintenance for the current year will be
Is. 3d. per week for pauper patients, that is 9d. a week less than the
preceding year; this sum includes the expense of clothing, and it is
believed that no lunatic asylum in the kingdom can afford co maintain
their patients at a less rate. The amount is considerably below the
sum charged for the maintenance of patients in the county lunatic
asylums of Middlesex and Surrey."
The medical officers congratulate the visitors upon the satisfactory
condition of the institution. We glean the subjoined statistics from
the superintendent's Report of the Littlemore Asylum:?
" The number of patients on the 1st of January and on the 6th of De-
cember, 1850, were, respectively?
In January
("Males 11G
I Females 170
[Total 286
In December
f Males 141
| Females 199
340
" The total number of patients at present in this asylum from the two
counties, including the city and boroughs in union, is nearly equal, though
the numbers of the sexes differ?namely,
From Oxfordshire?Males 59 From Berks 77
Females 107 83
Total 166 160
538 LITTLEMOIIE LUNATIC ASYLUM.
"Of tlic Oxfordshire patients, eleven females above 70 years of age,
average 70 years ; and of the Berkshire patients, three females above the
same age, average 77 years. The greater mortality of the males, and the
accumulation of aged females, is illustrated by a contrast with the Hanwe
Asylum.
Hanwell \ Males umler 35 years of age' Fcmales>
( ? above 35 years of age, 310. ? 457
Littlemore I ^ales under ^ JTenrs of aSe> 105. Females, 105
( ? above 45 years of age, 70. ? 120
This comparison of the numbers at different ages in the two asylums sug-
gests the propriety of giving also the different results, as regards recovery
and death; Hanwell containing the greater number of aged, and the less
of those recently attacked.
" Per centage on average number resident.
Recoveries. Deaths.
Hamvell Total average of 18 years 0-20 9-87
Average of the year 1849 3-43 7-18
Littlemore.........Total average of 4 years 1.8-0 11-4
Average of tlie year 1850 17-1 0-3
" Among the tables appended to this Report is one of the recoveries and
deaths in this asylum; giving various averages and adding the average of
seventeen county asylums, being, with one exception, all that I could make
available from the printed Reports. It will be seen that the mortality
being equal, the average of discharges is much in favour of the Littlemore
Asylum; a result which is the consequence of the ready admission of
patients into this asylum in the earlier stages of their insanity.
" Amongst the discharges of patients as cured, have been some of those
who had been long resident in asylums. The accounts we hear of them
are generally satisfactory as regards those who have returned to cottage
life. In the union workhouses they have sometimes not found themselves
situated as they had hoped to be ; and it has been reported of some of
them that they have been disappointed and have become not easily
manageable.
" The average age of inmates on 1st January, 1850, was?
Males
Females 40
Total average 45yL
Increase by Admissions Males.. 40T3^-
Females
Total average 40T%-
Decrease Males 45T22
Females
Total average 44
Of which the deaths were Males
Females 45
Total average 40Ty
This Report is made up principally of carefully arranged and well
digested tabular statements, reflecting much credit upon the medical
officer of the establishment.
SUFFOLK LUNATIC ASYLUM. 539
The thirteenth Annual Report of the Suffolk Lunatic Asylum, under
the able management of the resident physician, Dr. J. Kirkman, next
merits our attention. From this Report it appears that?
" There have been admitted in the year eighty-two patients,?forty-nine
have been discharged cured, and twenty-nine have died, according to the
table as made up to this day. There has been very little variation in num-
bers from last year's Report, there being only two more admissions, five
more cures, and one death less. Of the admissions, nineteen have been
first attacks, and within three months ; nine within six months ; seventeen
from a year and upwards; and thirty-six repeated attacks. On the whole,
thirteen suicidal, twenty-one dangerous, and nineteen both dangerous and
.suicidal. Three have been admitted in a very exhausted state, and sur-
vived their removal only a few days.
" The mortality has sensibly decreased for the last three years, which
may be attributable in some measure to the improved condition in which
the patients generally have been admitted, as well as to those sanitary
alterations which we have gradually been enabled to make. The house
has been very healthy throughout the whole year, and there lias scarcely
been an instance even of accidental injury. There are twenty-three epi-
leptics, (who are always more or less liable to sudden and violent falls,)
but amongst these there has been nothing to record in the medical journal
beyond a trifle.
" The return of the house as to-day, December 17th, 1850, is as fol-
lows :?
Males. Females. Total.
Patients in tlie House 31st December, 1840 . . . 119 134 253
Admitted since  30 40 82
155 180 335
Males. Females. Total.
Discharged, Cured .... 21 28 49
? Relieved . . . x 2 1 3
? Removed ... 2 0 2
Died 17 12 29
? ? ? 42 41 83
In the House, this day .... 113 139 252
We extract the following passages from the Report:?
" Two men were brought in under the most rigid restraint; one had
been fastened to a board on his back for a day, as a pretended additional
security ; his ancles and wrists were very sadly hurt. The other was tied
down in a cart with bands of no ordinary strength. In neither case was
any kind of personal restraint apparently necessary; in both, temporary
excitement was overcome, and entire quietude obtained, by kind and
soothing care ; they recovered, and remained well: one left in June last,
after being under treatment a month; the other in July, after three
months.
" The last of these cases was particularly impressive. The patient was
a high-spirited man, of more than six feet two inches, with a fine open
countenance, splendid contour, and commanding person; he was suscep-
tible and amiable, and within a few weeks after his admission, was a
regular cricketer in our uninclosed field. When he went away full of
gratitude, one could not help the expression of sorrow that a man should
have been brought to these gates so fettered and bound, who looked as he
left them to belong to the genuine aristocracy of the earth.
540 CASES OF INSANITY.
" Though tlie cases of patients discharged cured, are, as the result of
successful treatment, of more marked interest than others, it is not to such
exclusively that attention is directed. We have to learn from the expe-
rience of every day, that unsuccessful cases and failures have their instruc-
tive lessons respecting the treatment of those 'who come under the arbitrary
distinction of incurables. Many of these patients are most interesting
characters, frequently under rough exteriors hiding very delicate minds.
To hear their sorrowful descriptions, to be told the painful recollections of
former years, and to mark the still high level of their sensibilities, though
harassed by disquietudes and torn by alarms, would furnish a daily voca-
bulary for the book of experience, in which there should not only be
daily entries, but to which daily reference should be made for guidance in
future.
" The nominal distinction of' incurable' should not lead to relaxation of
effort, nor should it suppress the encouragement of hope. However
increasingly unfavourable the continuance of the malady beyond a definite
although variable period maybe, favourable results do occasionally reward
untiring efforts to obtain them. We have only just closed an interesting
correspondence with a discharged patient who left perfectly well in 1847,
and has continued so in different situations. She had spent nearly seven-
teen years in this house, and at times was very violent. It was thought by
some friends that her removal was hazardous, and in their anxiety and
fear they sought her readmission, though she was quietly and comfortably
living with her mother. Their anticipated dread of relapse has not been
realized. On the loss of her mother she has been noticed by some kind
and philanthropic friends in London, and as she was on the point of
sailing for America to keep her brother's house, she sent an affectionate
farewell.
" Nothing can more fully repay the anxiety attendant on that class of
patients where suicidal tendencies exist, than the knowledge that they do
not only return home well, but that they remain well.
" A. B. had been a trusted and trustworthy servant in a family of some
influence, who were very much interested in her welfare. She was a pale
nervous person, setat. 27, the subject of occasional hypochondriasis. About
eight months before her admission into the asylum, she was noticed to be
more reserved in her manner than usual: this apparent absence and un-
easiness increased, till her case assuming more decidedly the character of
suicidal melancholia, she was brought here on the 26th February, 1850.
When about eleven years old she met with rather a singular accident;
walking along the road, her clothes became entangled in the wheels of a
passing van; it was heavily loaded, and she was dragged for several yards
between the body and the wheels of the carriage: her thigh was broken,
and one arm in two places : she recovered from these injuries, but was so
constitutionally shaken as to be more or less subject to nervous agitations
ever since. She never likes to refer to the accident, and when it is men-
tioned, seems melancholy and distressed, and under the influence of
despondency, expresses a wish that she had been then killed. At the age
of sixteen she had small-pox, a long and dangerous illness supervening;
and she was left a good deal marked by the pustules. She complained on
admission of ceaseless headache, want of sleep, and a ' weight of anxiety,'
as she said, on the inability to fulfil her domestic duties. She was treated
with the light diffusible stinrali and nai'cotics at night, the acetate of mor-
phia, in -? gr. doses, and put on a mild nutritious diet. There was no
sensible improvement for some time : she was always endeavouring to be
alone, and talked generally in a most desponding manner on religious sub-
jects, and suffered greatly from the conviction that she had sinned beyond
HULL LUNATIC ASYLUM. 541
mercy. She used tlie warm bath twice a week, and took the sesqui-
carbonate of ammonia with evident advantage. In April her health began
to improve, and her morbid impressions gradually to subside ; she walked
a little occasionally in the garden, worked more collectedly at her needle,
joined a female reading class, and became a very attached and affectionate
patient; and continuing to improve, to enjoy her food, to be cheerful in
the day, and to sleep well at night, she was discharged cured on the 17th
May. She had a heavy disappointment on her return home, from not
again being received into her former service ; but she bore it well, and
obtained another situation, from which she occasionally writes, to say that
she remains very comfortable. This case was interesting and instructive.
The patient was decidedly a pious girl; and these morbid impressions
were evidently the result of an abnormal state of body, the healthy work-
ing of the spiritual gradually returning with that of the natural functions.
' There is little hope,' says the late Dr. Cheyne, ' in placing divine truth
before a melancholic, or hypochondriacal patient, until the bodily disease
with which the mental delusion is connected is cured or relieved.' It is
here indeed that the great advantage of domestic religious instruction is
felt, that as the process of bodily relief gradually goes on, by a watchful
and judicious conveyance the mental progress may be as gradually pro-
moted."
Dr. Kirkman is entitled to the warm thanks of all the friends of
humanity for the untiring zeal, skill, and humanity with which he is
carrying out the great work entrusted to him, in the institution over
which he presides. It gratifies us to have an opportunity of thus
expressing our opinion of his labours.
Through the obliging courtesy of Mr. F. "VV. Casson, the medical
officer of the Hull Borough Lunatic Asylum, we are enabled to pre-
sent to our readers the Report of this institution for 1851. It has been
kindly forwarded to us in manuscript. It appears that?
"The condition of the asylum has been healthy, except during the
months of August and September, 1849, when the town of Hull was so
severely visited by cholera, four deaths then occurring from that intractable
disease. Nine cases occurred in all, particulars of which have been given
in a former Report, and do not require any further remarks. Two or three
slight cases of erysipelas appeared in July, 1850, but beyond these no
epidemic has shown itself.
Males. Females. Total.
On the opening of the Asylum, July 1, 1849, there
were, transferred from the Hull Refuge ... 08 ... 30 ... 74
Admitted between July 1, 1849, and December 31,
1850   35 ... 34 ... 09
Total . . 73 ... 70 ... 143
Males. Females. Total.
Discharged?Recovered . . 18 ... 17 ... 35
Relieved . . 0
? Not Improved 2 ... 2 ... 4
Died 9 ... 7 ... 10
29 ... 28 ... 57
Remaining in the Asylum, Dec. 31st, 1850 ... 44 ... 42 ... 80
NO. XVI. N N
542 HULL LUNATIC ASYLUM.
" Tlie ages of tlie transfers were as follow:?
Males. Females. Total.
Under 20 years of age 1 ... 1 ... 2
Between 20 and 30 years of age 3 ... 3 ... 0
? 30 and 40 ?  8 ... 11 ... 10
? 40 and 50 ?  13 ... 10 ... 23
? 50 and CO ?  8 ... 7 ... 15
? CO and 70 ,,  4 ... 3 ... 7
? 70 and 80 ?  1 ... 1 ... 2
Total . . 38 ... 30 ... 74
" The ages of those admitted between July 1, 1849, and December 31,
1850, were as follow:?
Males. Females. Total.
Under 20 years of age 1 ... 1 ... 2
Between 20 and 30 years of age 7 ... 5 ... 12
? 30 and 40 ? .   7 ... 11 ... 18
? 40 and 50 ?  10 ... 11 ... 21
? 50 and GO ?  3 ... 3 ... G
? GO and 70 ?  G ... 3 ... 0
? 70 and 80 ? ....... 1 ... 0 ... 1
Total . . 35 ... 34 ... G9
" The forms of disease of the recoveries were?
Males. Females. Total.
Mania 5 ... G ... 11
Melancholia 4 ... G ... 1.0
Monomania 4 ... 2 ... 0
Dementia . .  1 ... 2 ... 3
Dementia, with Epilepsy 1 ... 0 ... 1
Surly, morose   1 ... 0 ... 1
Surly, morose, with Epilepsy 1 ... 0 ... 1
Irritable, and excitable, without delusions .... 0 ... 1 ... 1
Delirium tremens .1 ... 0 ... 1
Total . . 18 ... 17 ... 35
" The recoveries, as above stated, have been 35, or 24*47 per cent, on
the total number under treatment during the 18 months; and 50'72 per
cent, on the number admitted subsequent to the 1st day of July, 1849. Of
those discharged, recovered, two males have been readmitted; one for-
merly an epileptic, whose fits were removed some time prior to his dis-
charge, and who returned to the asylum after a lapse of nearly five months,
not, however, as an epileptic patient. The second case was one of maniacal
excitement, who returned, after having remained at home about a year.
Both these men remain in the asylum.
"The average residence of the patients who recovered was between
three and four months.
" A considerable portion of the admissions since the 1st of July, 1849,
may be considered recent cases. On particular inquiries, however, being
made of the relatives and friends of the patients, it has been found that
many were afflicted with insanity prior to admission, during much longer
periods than the statements in the forms of admission indicate. This is
much to be regretted, and it cannot be too strongly urged upon all con-
nected with the insane, the great necessity of an early removal to some
asylum, insanity being a disease which if attacked at its onset, generally
proves as curable as most other maladies.
" This mode of treatment can be adopted in pauper cases without
sinister motives being suspected, it therefore behoves all connected with
HULL LUNATIC ASYLUM. 543
the insane poor to promote their recovery by this means, not only on
account of the poor unfortunate individuals themselves, but also as pro-
ducing beneficial results to their respective parishes in a pecuniary point
of view.
" In addition to the recoveries, two females were discharged, relieved;
two male criminal lunatics, not improved, and two females not mentally
improved, were committed to the care of relatives, on their undertaking
the necessary legal responsibilities.
"The seventy-four patients transferred, on the opening of the asylum, had
for the most part been long afflicted with insanity, the great majority being
incurably idiotic or demented, forty-three of whom had been resident in
the Hull Refuge during periods varying from three to twenty-seven years.
" The forms of disease of the patients admitted between the opening of
the asylum on the 1st of July, 1849, and the 31st of December, 1850, were
as follow?viz.
Males. Females. Tctal.
Mania 7 ... 0 ... 13
Dementia   8 ... 10 ... 18
Monomania 5 ... 5 ... 10
Melancholia ?. . . . f) ... C ... 11
Dementia, with General Paralysis 3... 2... 5
General Paralysis 1 ... 0 ... 1
Idiots  2 ... 1 ... 3
Rambling, incoherent conversation 1 ... 2 ... 3
Irritable and excitable, without delusion .... 0 ... 2 ... 2
Surly, morose, with Epilepsy 1 ... 0 ... 1
Dementia, with Epilepsy 1 ... 0 ... 1
Delirium tremens .   1 ... 0 ... 1
Total . . 35 ... 34 ... 69
" The causes of insanity in the above being?
Males. Females. Total.
Loss of property 2 ... 2 ... 4
Loss of employment   3 ... 0 ... 3
Intemperance 4 ... 0 ... 4
Disappointment 2 ... 1 ... 3
Puerperal 0 ... 5 ... 5
Religious study 3 ... 0 ... 8
Hearing of many deaths from cholera 0 ... 2 ... 2
Over-exertion 0 ... 2 ... 2
Injury from fall 3 ... 0 ... 3
Diarrhoea 1 ... 0 ... 1
Constipation 0 ... 1 ... 1
Weak physical condition 0 ... 1 ... 1
Highly nervous constitution 0 ... 1 ... 1
Anxiety 0 ... 1 ... 1
Husband's long illness   0 ... 1 ... 1
Drunken husband 0 ... 1 ... I
Wife's misconduct   .... 2 ... 0 ... 2
Insufficient food 1 ... 0 ... 1
Cessation of Epilepsy 1 ... 0 ... I
Long Chancery suit   1 ... 0 ... 1
Brain fever 1 ... 0 ... I
Deficient development of brain   2 ... 0 ... 2
Unknown 8 ... 13 ... 21
Total . . 35 ... 34 ... CO
" The treatment adopted in each case cannot be given in a limited
Heport. General bleeding, as a curative means of insanity, Las been
N N 2
544 TREATMENT OF INSANITY".
adopted in one case only, that of a strong, powerful man, subject to out-
breaks of great violence. Large doses of opiates, combined with altera-
tives, tonics, (especially quinine,) purgatives, counter-irritants, &c., and
generous diet, have been employed in most of the cases of mania. The
great utility of the last?viz., good diet, was remarkably shown in the case
of a poor emaciated old woman, who came in a state of raving madness,
which, to all appearance, threatened soon to terminate her sufferings in
death. Immediately after her admission she was ordered porter and
plenty of nutritious food, by which means alone she was perfectly restored
to a state of sanity, and enabled to return home thirty-three days after
her arrival at the asylum. The treatment of other cases has been varied
according to symptoms. Two epileptics have been cured by the applica-
tion of a seton in the nape of the neck. Although one has been read-
mitted as a surly, morose lunatic, yet he has not had any return of the
fits for a period of eleven months. The second has been free from fits
during a longer period than this, although for many years prior to his
admittance into the asylum, he had been attacked with them almost
daily.
" The melancholies have been most benefited by regular employment;
and here I may remark upon the great utility of this as a remedial means,
in nearly all forms of insanity. Hitherto we have not had a sufficiency of
work; more, however, is being gradually introduced, and it is hoped, ere
long, that the whole of the inmates, with few exceptions, will be indus-
trially employed. A willingness should be shown, also, on the part of
the attendants, to use all the means in their power to induce the patients
to work, which may prove, perhaps, a little difficult to accomplish at first,
but which afterwards brings its reward in the greater quietude, order, and
contentment that is sure to ensue.
" No accurate account of the quantity or value of the work accom-
plished by the males has been kept. The appended statement, drawn up
by the matron, shows the amount of work that has been done by the
females.
" The number of male patients pretty constantly employed, and in what
manner, was as below:?
In garden 14
Assisting attendants, bed-making, scouring, &c. &c. . . 8
Painting, glazing, &c 1
In wash-house 2
In dry-house I
In laundry 1
Repairing clothes 1
Pumping 1
Cooking  1
30
" The mode of employment amongst the females was as follows:?
Sewing and knitting 16
In wash-house 5
In kitchen 1
Nurses' assistants 5
27
" Mechanical restraint has been sparingly employed; it has not, how-
ever, been altogether dispensed with, nor am I an advocate for its total
disuse, having witnessed its beneficial effects. In one instance it has been
wished for by a patient. One case only proved obstinate, and required
any lengthened restraint?viz., that of a man who ate his clothing, &c. &c.,
CASE OF SUICIDE. 545
indeed, anything he could procure, no matter of how filthy a description.
He was merely placed in a dress which gave all his limbs liberty except
his arms. It is singular that this man, when in bed, did not attempt
either to eat or destroy anything. Happily he has now relinquished his
revolting habit, and does not need any mechanical restraint whatever.
" The number of deaths during the year and half wa3 16, or 11*18 per
cent, on the whole number resident: a large mortality, it must be acknow-
ledged, yet deducting the four adventitious deaths from cholera the per
centage, viz., 8'39, is probably about the average of other English
asylums.
" The causes of death were as follow :?
Males. Females. Total.
Cholera 1 ... 3 ... 4
Natural decay 2 ... 0 .... 2
General palsy 3 ... 0 ... 3
Apoplexy 0 ... 1 ... 1
Epilepsy 1 ... 0 ... 1
Chronic disease of brain 1 ... 0 v.. 1
Congestion of brain 0 ... 1 ... 1
Phthisis . . . . ? 0 ... 1 ... 1
Shock, from a burn, on diseased nervous system . 0 ... 1 ... 1
Exhaustion from diarrhoea  1 ... 0 ... 1
Tctal . . 9 ... 7 ... 10
" The general health of the majority of the patients on admission was
good; there have been some lamentable exceptions, however. One man,
who came from a distance, had not been removed from his bed during a
fortnight before his admission. When brought to the asylum he had
bitten the first joint oft'his forefinger, and had an immense sloughing ulcer
extending over a considerable part of the back; he had an incurable
chronic affection of the lungs, and the lower limbs were paralyzed.
Altogether his condition was completely helpless. He improved during
the former part of his residence, but eventually died six weeks after
admission. A second male was sent from a short distance, in a dying
state. The whole of the limbs were completely paralyzed, and he was
quite unconscious. The breathing was indicative of the well-known
approach of speedy dissolution. He arrived on the afternoon of the
18th of August, 1849, and died early on the morning of the 21st.
" Such cases as the two above recorded help to swell the list of deaths.
" Both males and females assemble two or three times a week for an
hour or two before bed-time, for the purpose of dancing, some of the
patients playing the violin and flute. The attendants have remarked that
this has produced a beneficial effect; some of the inmates who are often
noisy and restless at night, being calm and quiet, and sleeping soundly
after having exercised themselves in this manner.
" One suicidal attempt only ha3 been contemplated in the asylum,
although thirteen males and fourteen females were stated to be dangerous
to themselves prior to admission. This solitary instance occurred in a
poor melancholy woman, who broke a window-pane for the purpose of
f>rocuring some glass, a portion of which (being of a triangular shape, in
ength 2? inches and in breadth If inch), by means of a piece of stick, she
forced down her throat as far as she could. My attempts to remove it
by instruments were ineffectual; I succeeded, however, in gradually
raising it higher by inserting my first and second fingers as far into the
gullet as possible; these endeavours were assisted by attempts at vomit-
ing, but so firmly was the glass embedded that it literally cut its way out.
Happily, this poor creature perfectly recovered, and, I believe, remains
well at the present time. She was one whose malady was produced in
546 BELFAST LUNATIC ASYLUM.
consequence of the shock upon her nervous system, by hearing of the
many deaths from cholera.
" It is very gratifying to hear of the continued welfare of those patients
who have left the asylum recovered. Some visit us, others "write. One
female, formerly a most violent and destructive person, but who is now
well and fulfilling some situation, frequently expresses herself as most
thankful for her recovery.
" The drainage has been materially improved, and is, perhaps, now as
efficient as it can be made on so flat a site.
" The weekly rate of charges is necessarily high for the maintenance
&c. of paupers, in a new asylum, many expenses being unavoidable at
first, which will not be incurred a second time. These charges, however,
were 2s. lower the first quarter, and Is. the five succeeding quarters, in
this asylum, than those of the North and East Hidings' Asylum, during
the first year and a half after it was opened, although it is at present
carried on at a less cost per head than any similar institution. Although
our expenses will decrease, yet it cannot be reasonably expected that the
reduction will take place to such an extent as where a considerably larger
number of inmates are received, as, of course, the greater the number,
the less will be the cost per head, and more especially so, if the industrial
plan is pursued, each individual by his work helping to reduce the amount
of his cost."
The Twenty-first Report of the Belfast District Asylum (1851) is a
very interesting and valuable document. The following statistical
information merits attention:?
General Statement of the Year's Admissions, &c.
Males. Females. Total. Males. Females. Total.
In Asylum, 1st April, 1850   147 121 268
Admitted since, new cases  68 66 134
? relapsed ditto .... 1 5 6
? ?   69 71 140
Total under treatment during the year  216 192 408
Discharged recovered, during the year . 33 48 81
? relieved ? . 11 18 29
Died, during the year  22 7 29
? ? ? 06 73 139
Remaining under treatment, 31st March, 1851 .... 150 119 269
Admissions this year more than last year  12 8 20
Daily average number of patients during the year 271-12
Do. for the year ending 31st March, 1850   267*50
Average annual expense of each patient this year, including every charge ?11 18 5
Do. for the year ending 31st March, 1850   12 17 3
Being a decrease on each patient this year of  0 18 10
Total expenditure for the year ending 31st March, 1851 . . . ?3232 2 0
Males. Females. Total.
" Dangerous Lunatics" admitted during the year, viz., from )
Antrim Gaol, 1 male, 1 female; from Down Gaol, 11 males, i 12 4 10
3 females   . . 1
"Dangerous Lunatics" in the house 31st March, 1851?viz.,) 6 1 7
from Antrim Gaol, 1 female; from Down Gaol, 6 males . J
" Criminal Lunatics" in the house 31st March, 1851 . . 3 2 5
The medical officers (Drs. E. Stewart, H. M'Cormac, J. S. Mul-
CRIMINAL LUNATICS. 547
Iiolland) speak highly of the Central Asylum at Dundrum, near Dublin,
for criminal lunatics. It is observed in the Report,
"It cannot be over-estimated the importance it is to these institutions
to be relieved from the ungracious charge of criminal inmates, and to
effect which, the governors of this asylum were the first to take up the
question, and unceasingly to keep it before the authorities, until ultimately
an act of the legislature was obtained, in 1845, empowering government
to establish an asylum exclusively for their due restraint and treatment,
and which, being now in operation, will, amongst other good effects,
remove the prison-like character which the district asylums sustained, by
being converted into places of incarceration for their confinement. And,
now that a precedent has been made by the founding of a criminal
asylum in Ireland, a general demand is making for one, also, in Great
Britain, which, for the welfare of such important institutions as the public
hospitals for the insane, will, it is hoped, be soon answered by placing
them on an equal footing, in this respect, with those in this country."
In these observations we fully concur. The following is satisfactory,
as showing the per centages of discharges and deaths, and the average
per centage, calculated on the average number of patients, for thirteen
years, ending 31st March, 1851:?
Years,
ending
31st
March.
1839
1840
1841
1842
1843
1844
1845
184G
1847
1848
1849
1850
1851
Yearly-
average
Number.
194-13
217-35
244-07
240-80
249-44
253-15
258-83
252-18
254-90
202-50
271-32
207-51
271-12
Recovered.
No. of
Cases.
58
55
04
72
90
09
08
01
00
81
09
50
81
Rate
Per Cent.
29-87
25-30
20-15
29-17
80-08
27-25
20-30
24-19
23-53
30-85
25-43
18-08
29-87
Relieved.
No. of
Cases.
7
5
9
11
13
13
21
14
22
15
14
22
29
Rate
Per Cent.
3-00
2-30
3-07
4-45
5-21
5-13
8-14
5-55
8.02
5-71
5-10
8-22
1009
Died.
No. of
10
28
24
27
18
21
40
24
27
47
30
43
29
Rate
Per Cent.
8-24
12-88
9-80
10-94
7-21
8-29
15-50
9-51
10-58
17-90
11-05
10-07
1009
Dr. Stewart is entitled to much commendation for his ably drawn up
Annual Report.
The Report of the West Riding of York Asylum for 1851 is before
us; and when we observe that it proceeds from the pen of Dr. Corsellis,
it will be sufficient to entitle it to every attention. The statistics of
the asylum are as follow:?
Males. Females. Total.
In the Asylum oil the 1st of January, 1850 . , 225 ... 207 ... 492
Admitted since  149 ... 130 ... 285
374 403 777
Males. Females. Total.
Discharged .... 59 ... 02 ... 121
Dead . 46 ... 35 ... 81 105 ... 97 ... 202
Remaining in the Asylum on the 31st of Decern- ?     !
ber, 1850   209 ... 300 ... 575
548 TREATMENT OF INSANITY.
Admitted.
Cnses not exceeding three months' duration, and first attack
Cases not exceeding twelve months' duration, and first attack
Gases not exceeding two years' duration, and first attack
Gases of more than two years' duration
Cases of those who have had previous attacks
103
43
11
50
Discharged.
Cases not having been insane more than three months before admission,
and discharged within six months 30
Cases not having been insane more than twelve months before admission,
and discharged within two years 27
Cases not having been insane more than ten years before admission, and
discharged within three years 8
Cases having had previous attacks 47
Cases not cured discharged by desire of their friends, and by order of
the magistrates 0
Males. Females. Total.
Admitted since the Asylum opened .... 2259 ... 2348 ... 4607
^Males. Females.
Discharged . 1002 .... 1331 ...
Dead ... 928 ... 711 ...
... 2042 ... 4032
Remaining  209 ... 300 ... 575
Males. Females. Total.
Number of Patients discharged cured . 878 ... 1001 ... 1939
? relieved 184 ... 270 ... 454
Average number of Patients during the Year, 554.
When speaking of the importance of continuous treatment, and the
folly often exhibited by relations in prematurely removing patients
from the control of asylums, merely because they appear calm, rational,
and are capable of work, Dr. Corsellis makes some sensible remarks.
He asks?
" How is it that society reason less correctly on insanity than on other
diseases ?
"The cerebral system is amenable to the same natural laws as other
parts of the human frame; and if, in other physical diseases, the moral
treatment forms a part, and no inconsiderable one, how much more
important is the careful and persevering use of curative means in that
class of maladies, in which the organ of thought itself becomes the prin-
cipal,seat of disease.
" Instances, not a few, might be adduced of relapses, in which the
patient has been brought back to the asylum, sunk in despondency and
self-renouncement, after having presented the most encouraging proofs of
convalescence, which might have been matured, had sufficient time been
allowed before old associations and former exciting causes had been again
encountered.
" To consider no case as hopeless, and to act with the best anticipations
in view for all, is as salutary a rule in assisting the ministrations and
labours of such as have charge of the insane, as it is difficult uniformly
and practically to carry out.
" Symptoms by which the disease is characterized are often so delusory
and capricious, their forms so changeful and indefinite, expectations are so
long unrealized, and efforts so tardily seen to produce any desirable effect,
CRIMINAL LUNATICS. 540
that the most practised observer may be at fault. In corroboration of
these remarks, a few cases, discharged in the past year, -will not bo
uselessly quoted, whilst they afford encouragement for future exertions,
and samples of a class of cases which might be more properly mul-
tiplied in another form of work, than in this annual report of a public
institution."
On the subject of suicide it is said??
" The two past years contain the record of no less than 133 patients-
admitted with suicidal propensity, suggesting the probability of epidemic
influence in this phase of mental disorder. From the month of June last,
seven females have been received, whose propensity to self-destruction
has been particularly declared in a determined resistance of food. With
a single exception, all were fed for a longer or shorter period, by the-
oesophageal tube; the resistance has given way, and, with the above
exception, they are progressing favourably."
"When speaking of criminal lunatics, the following appropriate
remarks are made?
""Whilst the enactments of a wise legislature have been directed to the
improved regulation of our system of prison discipline, so as to secure for
the culprit the best means of reformation, and for society the best
guarantee for protection; and whilst the lunatic, be he rich or poor, is
made the care of the State; it would seem inexplicable that a charge so
grave as that of criminal lunatics, one so irreconcilable with the harsher
features of prison appointments and with the mild governance and inse-
cure construction of asylums for the insane, should have been hitherto so
insufficiently provided for. The aggregate number of insane criminals in
the United Kingdom would surely warrant the construction of a suitable
separate building, in some remote part of the country, and the maintenance
of a duly qualified executive staff."
The following account of a Christmas gathering will be rj?ad with
interest:?
" Those of our unfortunate fellow-beings who labour under the most
severe affliction to which human nature is subject?the poor inmates
of the "West Hiding Lunatic Asylum,?have not been forgotten amid the
festivities of this joyous season. "We believe it has been for years past
customary, twice during the twelvemonth, to afford the harmless and
recovering patients, in this establishment, the means of exhilarating, and,
as far as they can appreciate it, rational, recreation. In the summer
season, when nature blooms forth in all loveliness and gaiety, they are
permitted and even encouraged to look forward with anxious expectation
for the day annually set apart for them to participate in the enjoyment of
healthful, out-door amusements. At Christmas, too, though necessarily
confined to a commodious apartment in the interior of the establishment,
a similar treat?so acceptable to these much-to-be-pitied mortals,?is
granted to them by the gentlemen who superintend the management of
the asylum. On both these occasions, one of the most interesting,
though at the same time, heart-depressing sights, is presented to the view
of the observant spectator. It is no less striking a scene than the
assembling and mixing together in orderly demeanour and quietness of
conduct, of more than two hundred fellow-creatures, cut off altogether as
it were from society, and exhibiting, more or less strongly, the varied and
to us innumerable forms which insanity assumes.?The concluding week
of 1850 was not allowed to pass over, without these poor people being-
550 A CHRISTMAS LUNATIC PARTY.
cared for as usual. The room iu which, they were regaled (if such an
expression be allowable,) at our Hiding Asylum, on Friday last, was
spacious and in every way suited to the purpose to which it was applied.
The spotless white walls were decorated tastefully with laurels and ever-
greens, displaying various devices; amongst which were most conspicuous
a representation of the Crown, with the letters Y. A. on either side,?
the initials of the title of this excellent institution, W. E. L. A., under a
wreath of evergreens forming the words ' God save the Queen,'?and the
three letters?C. C. C. The poor inmates were supplied during the day
with such provisions as their malady permitted; and in the evening par-
took of tea. The men, by far the smaller number of those present, with
the boys, were seated at one table, and the females at others; and they
sipped their refreshing beverage with the utmost order and discipline,?
those whose intellects were the least affected evincing deep attention to
the Grace sung before and after tea, as well as to the general proprieties
of the table. Seated here and there amongst this motley group, might be
selected many visitors who had experienced the benefits afforded by this
institution; and who, long after their recovery, would seem to make it a
practice to visit the establishment occasionally, and to offer such trifling
presents as are allowed, to relieve the tedious hours of those whose
malady is of a more severe and lasting character than their own had been.
Such acts of sincere kindness and gratitude are, it is represented, by no
means singular,?and this circumstance must afford high gratification
to every mmd which takes delight in bringing out and contemplating the
better and brighter lights, rather than the darker shadows, of human
nature. As may be well supposed, the patients varied as much in their
ages, their appearance, and their conduct, as they did in the degrees of
insanity. Here was to be seen the self-styled 'queen of the party,' a
portly, once-handsome woman, bedizened from head to foot with all the
gaudy finery and trinkets it was possible to heap upon her head and
dress,?seemingly gay and happy;?whilst, in another part of the room,
were presented to the wondering eye of the visitor, the slim figure and
graceful movements of a younger female,?her countenance vacant and
melancholy,?as she glided rapidly through the mazes of the dance, to
the exhilarating music of the piano, the violoncello, the violin, and the
flute, ? the latter instruments well played by inmates of the asylum.
Seated around the room were to be seen, in sad contrast, aged and youth-
ful idiocy,?viewing what was passing before their eyes, it is true, but
apparently unfeelingly and unconsciously,?some of the former amusing
themselves by examining a miniature doll of gaudy colours, or other fan-
tastical plaything,?the latter laughing listlessly, or moving restlessly
about. One there was, who now and then seemed unable to contain her
feelings, for she uttered occasionally a wild exclamation, and at once
resumed her calmness;?another looked indifferent and sullen;?and a
few?was it that 'the sound of sweet music made them sadP'?wept occa-
sionally and seemed relieved. None exhibited the least sign of dissatis-
faction or displeasure: whatever their mental afflictions, all looked con-
tentedly on the scene passing before them. The more ludicrous and
awkward the movements of the dancers, the greater the merriment, and
the louder the occasional clapping of hands by the patients, as a mark of
their enjoyment.?The evening, too, was not passed without several songs
being given by a recovered patient, in a manner little expected in such
a place. The 'Ivy green,' 'Woodman spare that tree,' 'Some love to
roam,' and others of the same class, were well sung in this strange party;
and numerous were the inmates who crowded around the vocalist, and
applauded his exertions! Nor should it pass unnoticed that several
DANGEROUS LUNATICS. 551
benevolent ladies and gentlemen mixed unreservedly in the pastimes of
the evening ? freely dancing with the grotesquely dressed inmates,
heartily joining in their choruses, and unceasingly administering, to the
utmost of their power, to their consolation and wants, by smiles, by kind-
ness, and by trifling presents. Nothing occurred to interrupt in the
slightest degree the pleasure of the evening: and at nine oclock, the
mixed heterogeneous company arose at a given signal, and remained stand-
ing whilst the National Anthem was sung; and then, the females pre-
ceding, the whole of the patients retired to their rooms with as much
silence and decorum as a devout congregation leaves a place of worship."
The Fourth Report of the Devon Lunatic Asylum (1850), contains
the subjoined statistical information :?
" During the past year 111 patients have been admitted; 52 have been
discharged, and 30 have died.
" The number of patients at the commencement of the year was 351; the
average number resident has been 372 ; and the number resident at this
date is 380; of whom 161 are males, and 219 are females.
" Forty-seven patients have been discharged recovered, and five have
been discharged relieved; of these 20 were men, and 32 were women,
being 46'8 per cent, on the admissions; the recoveries alone being 42"3
per cent, on the admissions.
" Of the patients who died, 17 were men and 13 were women, being 8
per cent, on the average number resident."
We direct the attention of the Lord Chief Baron Pollock to the fol-
lowing observations, in reference to that class of patients said to be
" not dangerous to themselves or others,"?
" This term I believe to be inapplicable to any insane person who is
not helpless from bodily infirmity or total loss of mind: it can only with
propriety be used as a relative term, meaning that the patient is not so
dangerous as others are, or that he is not known to be refractory or suicidal.
It should not be forgotten, that the great majority of homicides and
suicides committed by insane persons, have been committed by those who
had previously been considered harmless; and this is readily explained by
the fact, that those known to be dangerous or suicidal are usually guarded
in such a manner as to prevent the indulgence of their propensities, whilst
the so-called harmless lunatic or idiot has often been left without the care
which all lunatics require, until some mental change has taken place, or
some unusual source of irritation has been experienced, causing a sudden
and lamentable event. In an asylum such patients may truly be described
as not dangerous to themselves, or others, because they are constantly
seen by medical men experienced in observing the first symptoms of
mental change, or excitement, and in allaying them by appropriate remedies;
they are also placed under the constant watchfulness and care of skilful
attendants, and they are removed from many causes of irritation and
annoyance to which they would be exposed if at large, in villages, or even
in union houses."
The Report refers to a remarkable recovery which had taken place in
a female, aged thirty-six, who had been in a state of maniacal insanity
for twenty years: her recovery was gradual, and extended over more
than a year; she has been discharged five months, and her recovery
appears likely to be permanent.
552 INFLUENCE OF RELIGION ON THE INSANE.
The Reverend G. T. Lewis, the chaplain, makes the following judi-
cious observations in relation to the influence of religion on the
insane:?
"Apart from the benefits accruing, in a religious point of view, to the
insane, from the punctual observance of a routine of daily prayers, I
believe that such observance contributes, in an essential degree, to induce
habits of order and self-control, and is so far instrumental in maintaining,
if not in exciting, those moral influences, which, to so dependent a class as
those who have lost the guide of reason, are of incalculable importance.
Of my private ministrations at the asylum, I trust that I may say, with all
humility, that my intercourse with many of those patients, to whom my
attention has been specially directed, has been productive of good. Of the
admissions during the present year, a large number have belonged to the
class of those who are the subjects of acute religious melancholy. This
form of insanity is, at once, the most distressing, as well as obstinate and
capricious, of mental disorders. The difficulties which a clergyman has to
encounter, in his intercourse with those who are the subjects of this
dreadful malady, are various;?not the least of these difficulties is the
shrewdness with which patients of this description reason on their religious
state; supporting their distorted views by numerous, and, except in their
own cases, well-applied quotations from Scripture. To employ a process
of reasoning, in order to convince them that they are labouring under a
delusion, is unavailing, during the accession of the disorder, and is quite
as inapplicable to them as to any other class of the insane. I believe,
however, that much may be done in mitigation of the distress of these
afflicted people, by judicious reading, and by inspiring them with the idea
that you sympathise and take an interest in their state. This may be
effected by watchful and frequent visits, and by addressing them with con-
fidence and energy,?taking care, at these times, that the countenance,
which is scanned with impatient anxiety, betrays nothing of doubt or
despondency. But it is to the convalescent, of this as well as other classes
of the insane, that the visits of a clergyman are more especially useful, as
well as gratifying to himself. To .issist in re-establishing confidence,
where reason is returning, by animating and hopeful conversation of a
religious character, is, indeed, a pleasing, although delicate task. From
the majority of those who have been discharged during the present year,
I have received expressions of gratitude for the interest which I have taken
in their welfare."
Dr. Huxley's Report of the Kent County Lunatic Asylum (for 1849-
50) is extremely satisfactory. We make the following extract:?
" Fifty-six male and 60 female patients have been received, together
110, which, added to 383 remaining in the asylum at the end of the pre-
vious year, make a total of 499 under treatment in the whole period.
" Fifty-three men and 50 women, together 103, have been discharged or
have died; and 173 men and 223 women, together 396, remain.
"Twenty-five men and 21 women were discharged recovered, 5 of the
women having been first absent on trial when in an advanced stage of con-
valescence. These were all reported to have done well, and were then
absolutely discharged. One woman, still out on trial when the year
closed, relapsed, and has been brought back to the asylum. Two of the
men suffered speedy relapse, both being subjects in whom there was slight
probability of permanent freedom from insanity, on account of previous
attacks. Both had, however, recovered and remained well so long pre-
CRICHTON ROYAL INSTITUTION. 553
vious to their discharge, that there was no sufficient ground for their
detention.
" Twenty-six men and 23 women died of grave diseases which are, in no
small proportion, peculiarly fatal to insane persons.
" Fifteen of the patients admitted were suffering from repeated attacks.
These, compared with the remainder, form about an eighth part of the
whole.
" The rates of recovery and death have both been higher than in 1848-9.
The proportion of recoveries is 39"6 per cent, to the admissions, instead of
the 32'4 per cent, of 1848-9 ; that of the deaths is 12"7 per cent, on the
mean daily number (385'1), instead of the 10'5 per cent, of the previous
year.
" Within a fortnight of the close of the year twelve patients, besides
those ordinarily admitted, were received under circumstances which had
just extended the use of the asylum to them. In so short a period, these
could not contribute to the recoveries, the proportion of which, however,
they reduced by swelling the admissions. "Without these twelve, the rate
of recoveries would have been 44'23 per cent."
We copy a portion of the general statistical statement?
Males. Females. Total.
Remaining in tlie Asylum July 4tli, 1819 . . . 170 ... 21.3 ... 383
Admitted in the year ended July 1, 1850 ... 50
Patients under treatment during the year . . 220
Deduct numbers discharged and dead during tbe
vear 53
00 ... 110
273 ... 109
50 ... 103
223 ... 390
Remaining on the 1th July, 1850   173
Patients were discharged as follows :?
Males. Fern. Males. Females. Total.
Recovered 25 ... 10
On trial for a month, since
recovered 0 ... 5
? ? 25 ... 21 ... 40
Convalescent (still out on trial)  0 ... 1 ... 1
For removal to other Asylums   2 ... -4 ... 0
Not cured  0 ... 1 ... 1
Dead  20 ... 23 ... 49
53 ... 50 ... .103
The admissions consisted of?
Males. Females. Total.
Admissions for the first time 47 ... 54
Admissions repeated 9 ... 0
50 ..! 00 ... 110
Average daily number of patients resident throughout the year, 385*19.
Dr. Browne's Eleventh Report of the Crichton Royal Institution,
Dumfries, is more of the character of an essay than a report. It is an
able document.
When speaking of the moral condition of patients, and the delusions
of sound minds, Dr. Browne remarks?
" It is proposed that the moral condition of the individuals admitted
should be considered in reference to the presence or absence of delusion
554 DELUSIONS OF SOUND MINDS.
as an element of disease. Coleridge has said that society would be broken
up, that man would loathe his brother man, if the secrets of each heart
were laid bare to public gaze. It is certain that every heart has some-
thing to conceal; a sorrow, a sin, or a folly. To affirm that there is some
dark passage, some spot of soil and shame, some tyrannous passion or pre-
judice, in the history of every life, may appear but another form of the
truism, that to err is human. But it is not suspected that so many minds
endowed with robust and splendid qualities cherish some wild and baseless
belief, are haunted by superstitious fears, or are the unresisting victims of
delusion. The confessionals of medical men, however, declare the fact,
that the presence of signal and unequivocal eccentricity and hallucination
is compatible with the exercise of sound judgment and brilliant fancy, with
the faithful discharge of vast responsibilities, and with the external cha-
racteristics of perfect sanity. The calm, contemplative mathematician and
satirist, Pascal, rested for years on the brink of an imaginary gulf: the
adventurous warrior who hewed his way to the throne of Sweden was
daunted and diverted from his stern purpose by an apparition in a red
cloak. Extreme cases are recorded where men have been accompanied by
a skeleton step by step of their course; where a gory head has crossed the
gaze of the impassioned orator; where one horrible thought recurring
periodically has haunted its victim to despair and death ; but instances are
constantly met with where individuals carry into ordinary intercourse and
active life tendencies to destroy children, grotesque convictions that their
frame is tenanted by unclean beasts, that they are infected by foul diseases,
that their passions are acted upon by the will of others, and extravagant
fancies . that the future is opened up to them, that they enjoy com-
munion with unseen beings, that they see, and hear, and deal with
objects hidden from common observation. In such circumstances, the
mind either detects the true nature of the impression, knows that it is dis-
eased, refuses credence to the morbid suggestion, and struggles with and
subdues the tendency; or, attributing these to errors of sense or external
circumstances, it disregards their influence; or, separating them from its
ordinary operations, it is partially affected, but acts independently of their
presence; or, receiving them as realities, there remains the prudence to
conceal, although there is wanting the wisdom to resist. To the latter
condition may generally be traced those instances of eccentricity and pecu-
liarity which seem to be without cause, and inconsistent with the tenour of
the character upon which they are engrafted. The eccentric man is pitied
or persecuted. He is excluded from society as a bore, or admitted as a
butt. He is condemned as ill-educated, as regardless of the comforts of
others, and indifferent to their censures. It would be censorious to adopt
the opinion of Mackenzie, that ' delusive ideas are the motives of the
greatest part of mankindbut it would be a humane and correct philo-
sophy to trace their absurdities to diseases, to recognise in their extrava-
gance and contravention of all established rules and customs, the exhibition
of a deep-seated delusion, which may fetter attention or obscure memory,
while it leaves the judgment free and the affections warm and faithful.
Newton forgot the brief portions of time which separated his meals in the
calculation of' cycles in epicycles rolledand to the impoverished and
enfeebled mind the contemplation of Napoleon's hat in the sun may be as
engrossing a topic."
We have only space for one more extract. It refers to the subject of
moral insanity?
" Crime and insanity often meet and mingle. Many of the horrible
CRIME AND INSANITY. 555
tragedies -which disturb society ma}' be the result of such a combination.
They may be the natural manifestations of disease engendered by, or
associated with, dissolute habits, brutal appetites, and violent passions.
Observation has proved that a large proportion of criminals are of imbecile,
contracted, and depraved intellect; that they are subject to delusions;
and it is equally established that the insane are less regulated by con-
science and religion, less restrained by law, and custom, and opinion, than
those unaffected by disease. But there is a class of persons who cannot,
in the ordinary sense, be regarded either as insane or culpable, but who
are unquestionably of unsound mind, who, with moderate intelligence and
cultivation, and in favourable circumstances, commit acts which outrage
decorum and virtue, which are inconsistent with the knowledge and posi-
tion of the perpetrator, which are subversive of the best interests of the
individual and the community, and which, although voluntary, deliberate,
and avowed, evidently flow from perverted affections and debased propen-
sities ; and wThich, temporarily at least, obscure if they do not suspend the
influence of the judgment, moral sense, and selfish considerations. The
frenzy or feebleness of the common phases of insanity are readily reco-
gnised ; but it is difficult to trace in the recklessness of the spendthrift, in
the excesses of the voluptuary, or the callousness and cruelty of the de-
bauchee, the fruits of disease, to admit as moral insanity what appears to
be moral turpitude. That derangement does affect the sentiments is shown
when the whole mind is involved in general mania, and the dictates of con-
science are as absurd as those of reason; but while history abounds in
illustrations of this form of disease, it has only recently been suggested
that the emotions and passions might be subject to special disease, might
be affected independently of the intellect, and while all the other faculties
remained apparently active and unimpaired. The conclusion was forced
upon observers by the occurrence of cases totally irreconcilable with any
known species of insanity, of children nurtured with care, and circumspec-
tion, and prudence, growing up, in defiance of all tender and virtuous
influences, ruffians and desperadoes; of men of polished manners and
refined tastes delivering themselves up to the indulgences of furies or
felons, of causeless and inexplicable atrocities, of loathsome and revolting
practices. It is probable that in every case of this kind actual disease
will be found superadded to original defect; that a change of character,
or temper, or taste, originating perhaps in bodily infirmity or degenera-
tion, will be discovered in conjunction with original peculiarities of mental
constitution; that while the capacity of the mind was enlarged, its self-
control was neglected; that while the perception of right and wrong was
present, the feeling of moral obligation was defective. Moral insanity
may be impulsive. The morbid tendency may arise suddenly, strongly,
irresistibly, and precipitate the actor into a course diametrically opposed to
his previous conduct and character; or, it may be the conclusion and
completion of a series of irregularities. A passion may be nursed and
nourished until it obtains dominion over every other power; or, thirdly,
tendencies in themselves diseased and hideous, long subdued by reason or
religion, or disguised by prudence, are developed by the decay and dete-
rioration of better principles, by external temptations ; or, fourthly, the
moral sense is weakened or warped, in the same manner as the will or the
imagination, by cerebral disease. To this last category are many of these
examples of ostentatious depravity, or grotesque vices, to be referred,
which occur after middle age. The amount of disease may be so slight as
to have produced little impression upon the vigour of the constitution, as
to have escaped the attention of the sufferer, as to have occurred from a
blow or a fall, or in the congestion of fever, or in delirium tremens, or from
-55G MORAL INSANITY.
those changes which luxury or habitual cxcitement or age seem calculated
-to produce ; but still, it may be capable of modifying the disposition, and
affecting every law and association of the mind. How far such elements
should be allowed to enter into legal investigation may be doubtful; but
in all medical inquiries as to sudden or otherwise inexplicable changes of
temper or tendency, the fact should never bo forgotten that they are
symptoms of the structural alterations in apoplexy and congestion. To
such an origin will it be incumbent to attribute a case recently admitted,
where, with great natural shrewdness, general information, and gentle-
manly manners, where no delusion or incongruity of thought can be
detected, there exists an inveterate desire to torment and irritate those
around, to enjoy the dissension and disputes which ensue, and to violate
every rule of decency and delicacy by obscenities of look, word, and action,
when these objects can be accomplished without detection. These qualities
render the presence of such a person in society or in a family a nuisance
and a poison. Viewed alone they must be stigmatized as vile and vicious ;
viewed in relation to the coexisting powers and habits, they are inexpli-
cable ; but viewed as a part of the physical history of the individual, as
consequent on a period of violent excitement, an attempt to destroy life,
and an attack of melancholia, they take their place as indications of con-
ditions affecting the whole system, and among phenomena over which the
ivill possesses imperfect control."
The Second Annual Report of the North Wales Lunatic Asylum,
((Denbigh,) 1850, is before us. "We are glad to hear that the institution
is in so satisfactory a state. We copy the following table, showing the
admissions, discharges, and deaths, through the year.
In the House ^durhiirD Discharged? Remain-
Jan. 1, 1850. tjle yea^. Cured. Improved. Unimproved. Died. ing.
?Private?males 3 11 3 2 2 ... 7
? females 4 4 ... 3 1 ... 4
-Paupers?males 43 31 9 1 ... 5 59
? females 57 30 10 1 ... 5 65
Total . . 107 76 28 7 3 10 135
The annual receipts of the asylum were 58071. 17s. 6d.
We copy from the Fourth Report of the Lunatic Asylum for the
North and East Ridings of Yorkshire, 1851, the following statistical
data :?
Males. Females. Total.
There were in the Asylum on the 1st January, 1850 ... 81 ... 78 ... 159
Admitted to the D 1st December, 1850   9 ... 9 ... 18
90 87 ... 177
Discharged cured 1 ... 7 ... 8
Removed, chargeable elsewhere, or at the request of friends,
being no longer chargeable 3 ... 1 ... 4
Died  8 ... 3 ... 11
Remaining in the Asylum on the 31st December, 1850 . . 78 ... 70 ... 154
" Of the 177 patients under care, 6*21 per cent. died. The daily average
number in the house was nearly 160, of whom 6'875 per cent. died.
"From the opening of the asylum, on the 7th April, 1847, there have
been admitted 154 males and 130 females, together 284 patients; of
whom, 31 males and 9 females, total 40, are deceased; and 40 males and
QUESTION OF RESTRAINT. 557
43 females have been cured, total, 83; of this number 5 males and 4
females have been re-admitted. Two of the re-admitted males and two
females have been twice discharged. One of the males and two of the
females remain, and two of the re-admitted males are included in the
obituary. Consequently, 76 of those recovered, namely 37 males and 39
females, continued well up to the 31st December last.
" Calculating the cures for the year, upon tbe admissions for the same
Iieriod, it will be seen that 44i per cent, were discharged. With the
amentable fact that out of the 18 patients received in 1850, only one
female presented a fair hope of recovery, and that the others were afflicted
with chronic mania, idiocy, and epilepsy, it is obvious that the per-centage
of cures mainly depended upon the restoration of some of those admitted
in former years. In last year's report it is stated, that out of 159 patients
remaining in the asylum on the 31st December, no less than 149 were of
the unfortunate class considered incurable; leaving only 10 cases of a
hopeful character, which, together with the one mentioned above, made a
total of 11 curable patients; of whom 8 were actually cured and dis-
charged, thus showing the cures upon that class to amount to upwards of
72 per cent."
We direct the attention of those who take an extreme view of the
question of restraint, to the subjoined remarks:?
" The violence of madness may, perhaps, be overcome by physical
strength in a workhouse, but there will be an absence of that medical and
moral influence exercised by those who are familiar with the insane, and
which operates so wonderfully upon their conduct. Reference may here
be made to the so-called non-restraint system of management; the advo-
cates and promulgators of which system?according to my view scarcely
intelligibly named?admit the necessity of occasionally restraining some
violent lunatics, which they prefer doing by means of the attendants laying
hold of them, than by the employment of anything to be placed upon the
maniac's person. This much-boasted system is doubtlessly recommended,
as being more merciful than the use of old-fashioned manacles, leg-locks,
strait-waistcoats, &c.; but I apprehend that padded rooms and super-
human-like attendants?if they can be procured?cannot honestly be said
to entirely supersede, in all eases, the use and aid of strong dresses. One
might go further in explanation of my meaning, and state that seclusion
in a padded or single room, is only another kind of restraint. And that
so long as the separation of one lunatic from another is found, under cer-
tain circumstances, to be a salutary, safe, and requisite mode of treatment,
and that the physical energies of the attendants are needed to prevent the
desperate attempts which some of the insane inhabitants of an asylum
make upon themselves or others, or to check and arrest the mischief done
to clothing, bedding, furniture, fittings, &c., just so long will a non-
restraint system of treatment for the insane be one only in name."
When speaking of the advantages of employment, as a curative agent,
the following sensible observations are made:?
" In awarding to industry the highest place amongst the moral agents
for the cure and treatment of insanity, let me not be understood to disre-
gard cheerful recreations and pastimes, and occasional meetings of a plea-
surable and innocent kind, as important and necessary auxiliaries to a
community like the inhabitants of a lunatic asylum. Rational enjoyment
is very desirable to assist in dispelling or keeping in chcck the mental
harass to which they are so painfully subject.
NO. XVI. O O
558 ADVANTAGES OF EMPLOYING THE INSANE.
In the above remarks we fully concur. None but those experienced
in the treatment of the insane are competent to appreciate the difficulty
the physician has in systematically employing them. In public asylums
the matter is of easier accomplishment, because nearly all the patients
have been accustomed to work manually for their daily bread; but in
private establishments the case is very different. The musician may
have his favourite instrument.?the literary man his books,?the painter
his palette,?but alas! all former habits and tastes are often annihilated,
and it is often more than useless, in fact, irritating, to press occupation
upon them, until the malady has partially yielded to medical treat-
ment. Occasionally the first indication of returning health is a volun-
tary wish for some employment.
" To reclaim the disordered mind from bewilderment, to divest it of
torturing thoughts, to bridle the incoherence of the loquacious, to dissipate
the imaginary ailments of the hypochondriac, to cheer the dispirited and
sad, to give hope to the fanatic, to bring within possible limits the aspira-
tions of the exalted and extravagant, to inspire with confidence the mind
void of such an attribute; to effect all these, and the many other wants of
an asylum life, every expedient wliich humanity can suggest, or ingenuity
devise, should be brought into the category of remedial agents. Perhaps
one successful example is better than a dozen pages of theory. The
patient whose case I will narrate, was admitted from another asylum,
wherein opportunities for employment did not exist. He was associated
with some eight or ten other lunatics, in different conditions of insanity,
varying from mania to established dementia, and was confined in a day-
room which opened into a small airing court surrounded by high walls.
His appearance indicated melancholia, which, upon a careful scrutiny, was
found to arise from a belief that he had not 'a spirit like another man,'
that 'he ought never to have been born,' and that those who had given
him origin were amenable for his misery and suffering. Impressions of
such a nature have led to disasters involving the commission of double
crime. Such a tendency caused much anxiety. The principles upon which
suicidal patients are managed in this asylum, were explained two years
ago. This man was a blacksmith by trade, and he was, therefore taken to
the blacksmith's shop, where he immediately commenced working. The
influence which novelty of position exercises over the mind of the insane
is often very astonishing. In this case, another kind of responsibility was
assigned to him, besides those of the forge and anvil: an unridy patient
was set to work in his company, whose propensity to steal and frequent
attempts to escape, besides some other objectionable practices of which he
was guilty, rendered it indispensable that he should be narrowly watched;
this trust was faithfully kept by the blacksmith patient throughout his
sojourn in the asylum. A lathe soon afforded him another novelty, with
which he became perfectly fascinated, although he had never previously
handled a turning tool. An inventive genius soon manifested itself, which
prompted him to contrive a back-action lathe of almost unique construc-
tion; he also became a proficient in making screw-stocks and dies.
Nothing could be more striking than the salutary effects of these various
occupations. To use his own expression, he said, ' I am in heaven now
compared with what I was, and I am sure I should have become an idiot
had I remained where I was.' Since his discharge he has given practical
proof of the gratitude he feels, and is now an intelligent and useful member
of society, living in the bosom of his family."
GLOUCESTER LUNATIC ASYLUM. 559
The Report of the County Lunatic Asylum, Gloucester, 1850, con-
tains the following table:?
Remaining in tlie House Dec.
31, 1849
Out on trial
Admitted during tlie year
Re-admitted ditto
Total under treatment in tlie
year
Discharged:?
Recovered and gone . . .
Ditto, out on trial . . .
Ditto, Relieved
Not Relieved, removed by
friends
Died
Total . . . .
Remaining in tlie House Dec.
31, 1850
1st Class.
M. F.
9
2nd Class.
M. F.
19
23
.15
1G
20
15
3rd Class.
M. F.
110
147
111
138
1
199
151
M.
13G
1
179
50-
130
1G0
1
29G
298
91
15
225
54
170
404
104
300
We glean the subjoined facts from the Report of the Northampton
General Lunatic Asylum, 1850:?
" 102 patients have been admitted into the house; of whom 32 were
private, and 70 paupers.
" 73 have been discharged; of which number 64 were recovered or
greatly relieved; whilst 9 were transferred to other institutions, unim-
proved.
"34 have died during the year, being about 12? per cent, on the daily
average number of 264. The mortality table thus exhibits a higher range
than for the previous year; but a careful inspection of it shows that no
less than 12 of the year's admissions were persons either of advanced life,
or otherwise labouring under severe derangement of the vital organs; 4
were bed-ridden from the commencement of their residence until their
death. The average age of death seems to be 48."
We are glad to hear that this institution has enjoyed a happy
immunity from all disease of an unusual or epidemic character.
It appears from the last Report of the Royal Edinburgh Asylum for
the Insane, that the average number of patients, in all departments,
during the year, was 497?being 24 more than in the year preceding.
The amount of ordinary receipts by the treasurer,) , ,r.c
during the year, was j ?14,103 19 11^
And of his disbursements     12,193 G 2?
Thus leaving a surplus income of  ?1970 13 9
o o 2
5G0 HOMICIDAL INSANITY.
The following table, which we extract from Dr. Slcae's valuable
Report, will give our readers a correct idea of the present condition of
this excellent institution:?
Number of inmates at tlie close of 1849
Admitted during the year 1850 . . .
Total number under treatment . . . .
M. F. T.
Discharged . . 78 88=1GG
M. F. T.
Of wliom were cured . . 47 04=111
,, uncured . 31 24= 55
Died    20 38= 04
Total number at the close of 1850
Males.
224
.120
350
104
240
Females.
251
127
378
120
475
253
728
230
498
Average number resident during the year 1850:?
Males, 241*5. Females, 255*0 Total, 497*1.
" The number admitted during the year (253) is twelve less than there
were the previous year, but the average number residing in the house
(497) is considerably greater. In 1849, the average number resident
was 473.
" The number of patients discharged cured was 111, being in the ratio
of 43 9 per cent to the number of admissions, and of 22*65 per cent, to the
mean number resident.
" The total number of patients admitted into the asylum, since its
foundation, is 2432. The number dismissed cured is 989,?being in the
ratio of 40*6 per cent, to the whole, or 51*1 per cent, deducting those still
under treatment."
On the subject of moral insanity and homicidal impulses, it is observed:
" Of the four cases of moral insanity included in the preceding table,
one presented some features of peculiar interest, in a medico-legal point
of view. It was that of a female, labouring under a powerful homicidal
impulse. She had no disorder of the understanding, nor perversion of
her intellectual powers,?and, in particular, she laboured under no delu-
sions or hallucinations. She had a simple abstract desire to kill, or
rather, for it took a specific form, to strangle. She made repeated
attempts to effect her purpose, attacking all and sundry, even her own
nieces and other relatives,?indeed, it seemed to be a matter of indif-
ference to her who she strangled, so that she succeeded in killing some
one. She recovered, under strict discipline, so much self-control as to be
permitted to work in the washing-house and laundry, but she still con-
tinued to assert that she ' must do it,' that she was ' certain she would do
it some day,' that she could not help it, that ' surely no one had ever
suffered as she had done,'?was not hers ' an awful caseand, approach-
ing any one, she would gently bring her hand near their throat, and say
mildly and persuasively, ' I would just like to do it.' She frequently
expressed a wish that all the men and women in the world had only one
neck, that she might strangle it. Yet this female had a kind and amiable
disposition, was beloved by her fellow-patients, so much so that oue of
CHESHIRE AND YORK LUNATIC ASYLUMS. 6GI
them insisted on sleeping with her, although she herself declared that she
was afraid she would not be able to resist the impulse to get up during
the night, and strangle her. She had been a very pious woman, exem-
plary in her conduct, very fond of attending prayer-meetings, and of
visiting the sick, praying with them, and reading the scriptures, or
repeating to them the sermons she had heard. It was the second attack
of insanity. During the former she had attempted suicide. The disease
was hereditary, and it may be believed that she was strongly predisposed
to morbid impulses of this character, when it is stated that her sister and
mother both committed suicide. There could be no doubt as to the
sincerity of her morbid desires. She was brought to the institution under
very severe restraint, and the parties who brought her were under great
alarm upon the restraint being removed. After its removal, she made
repeated and very determined attacks upon the other patients, the
attendants, and the officers of the asylum, and was only brought to exer-
cise sufficient self-control by a system of rigid discipline. This female
was perfectly aware that her impulses were wrong, and that if she had
committed any act of violence under their influence, she would have been
exposed to punishment. She deplored, in piteous terms, the horrible
propensity under which she laboured."
We regret that the waut of space prevents our making further
extracts from this valuable Report.
The Report of the Cheshire County Lunatic Asylum, presented in
April, 1850, gives the subjoined particulars:?
Males. Females. Total.
77 .. 97 ... 174 ) .0,
Admitted from 1st Jan. to tlie 31st Dec., 1849 .41 ... 30 ... 71
There have been discharged?
Recovered 8 ... 14 ... 22
Relieved 10 ... 9 ... 19
Not improved  3 ... 1 ... 4
Escaped  1 ... 0 ... 1
22 24 40
Died  . . 7 3 10
56
Leaving in the liouse, males 89, females 100?Total . . . 189
The following extracts will convey to our readers an accurate idea of
the condition of the York Lunatic Asylum, as presented in the Report
for the year ending June, 1851;?
Monthly average number of patients in the house, from June,
1850, to June 1851 141
Patients admitted from the first establishment in November,
1777, to October, 1814   2035
Discharged cured, improved, and removed by their friends (the
proportion of each not ascertainable) 2133
Died 399
Remaining in the asylum, October 10, 1814 103
2G35
Patients in the asylum, October 10, 1814 103
Admitted from October 10, 1814, to June 1, 1850...1555.7 , ,Qr
?To June 1, 1851...40 Total}
1098
562 STAFFORDSHIRE LUNATIC ASYLUM.
Dischabged.
From October 10, 1814, to June 1, 1850. To June 1, 1851. Total.
Cured  558 ... 18 = 576
Improved 318 ... 15 = 333
Removed by their friends. . 310 ... 11 = 321
Died  335 .. . 8 = 343
1573
Remain in the House.
June 1, 1850. June 1,1851.
WoLk! C*|137 Women II | 135 ~ 15,3 = 1088
J. W. Metcalfe, Resident Medical Superintendent.
Dr. Flynn's Eeport of the District Lunatic Asylum, Clonmel, is
extremely satisfactory. By it there appears,
Males. Females. Total.
Remaining in asylum on 1st April, 1849 . . . 64 ... 69 ... 133
Admitted up to 31st March, 1850   19 ... 11 ... 30
Males. Females. 83 ... 80 ... 163
Discharged cured 11 ... 13
Not cured 1 ... 1
Died 7 ... 2
19 ... 16 ? 19 ... 16 ... 35
Remaining on 1st April, 1850 .... 64 64 128
Per-centage of cures on admission 80 per cent.
Per-centage of cures on average, in asylum .... 19 per cent, nearly.
The general result of the year -will appear from the following table
attached to the last Eeport of the Lunatic Asylum of Aberdeen:?
Males. Females. Total.
Patients in the asylum, 1st May, 1849 . . . 119 ... 107 ... 226
Admitted during the year  36 ... 45 ... 81
Under treatment during the year  155 ... 152 ... 307
Removed during the year?viz.
Males. Females. Total.
Recovered . . 15 ... 21 ... 36
Improved . . 5 ... 12 ... 17
Unimproved . 3 ... 5 ... 8
Dead .... 12 ... 4 ... 16 ? 35 ... 42 ... 77
Remaining in the asylum, 1st May, 1850 . . 120 ... 110 ... 230
The following is a general statement of patients admitted, discharged,
and now on the books, from the opening of the Staffordshire General
Lunatic Asylum, October 1st, 1818, to December 31st, 1849:?
Total number of admissions  3424
Discharged recovered  1504"1
Ditto relieved 471 I
Removed, as harmless or incurable, or by desire of friends . . 490 f
Died  713 J
3178
Remaining under cure 26 )
Ditto incurable  220 )
246
ADVANTAGES OF EARLY TREATMENT. 563
The subjoined extract is a general statement of patients admitted,
discharged, dead, and remaining on the books, from the opening of the
Nottingham Lunatic Asylum, on the 12th of February, 1812, to the
31st of December, 1850:?
Total number of cases admitted?males 1073
? females  897 _ 1970
Cases of re-admission? males 19G
,, females 145 ? 341
Discharged recovered
Ditto relieved . .
Ditto not relieved .
Dead
Remaining, Dec. 31st,
considered curable
Considered incurable
Total admission . . . 2311
. . males 540
females 520 ? 1000 total recovered.
. . males 254
females 199 ? 453 ? relieved
. . males 113
females 79 ? 192 ,. not relieved.
. . males 242
females 12G ? 3G8 ? dead.
1850, ) males 10
) females 12 ? 22 ? remain incurable.
. . males 113
females 103 ? 21G ? remain incurable.
2311 Total general treated, 2311
"We call the attention of our non-professional readers to the following
passage, extracted from the Report of the Liverpool Lunatic Asylum
for 1850, pointing out the importance of early treatment:?
" In the first place, it is painful to observe that in too many instances
removal from home has been delayed until the prospects of cure are almost
hopeless. A slight deviation from the patient's usual deportment is
observed: this is perhaps attributed to some physical disorder with which
it may be allied: the medical adviser is called in: he shrinks from the
responsibility of recommending the patient's removal to an asylum, until
the state of mental alienation is such, that further delay is unsafe; and
then it not unfrequently happens that the patient is hopelessly insane.
The friends are usually governed by their medical attendant: still they
desire, if possible, to avoid the necessity of removing their dearest rela-
tions from their own immediate care. This difficulty is not felt by those
whose friends have been previously confined: they usually act with promp-
titude, and save themselves and the patient much unnecessary trouble and
distress.
" In one case admitted, the patient had been confined in a small room
for five weeks, and subjected to the incessant torture of a straight waist-
coat. His mind had become so irritated, and his bodily health so enfeebled,
that it was considered dangerous to remove him. He was nourished by
generous diet, and, being exceedingly mischievous, irritable, and disposed
to suicide, was carefully watched; he improved rapidly, and at the end of
three months he left the institution well in body and mind."
The subjoined table is given, with a view of furnishing statistical
information on the subject to which it refers, showing the admissions,
re-admissions, discharges, and deaths, during the year 1850.
564 TREATMENT OF EPILEPSY.
Remaining in the Institution, 1st January, 1850 ,
Admitted for the first time during M. F. T.
the year 10 10 20
Re-admitted during the year . . 3 3 0
Total admitted
Total under care during the year . . . .
Discharged:? M. F. T.
Recovered S 7 15
At friends' request, relieved .35 8
At friends' request, not im-
proved  4 2 0
Removed to County Asylum ..10 1
Dead 0 5 11
Males.
32
19
Total discharged and died during the year
Remaining in the Institution, 1st January, 1851
Average weekly number in the house . . .
51
22-
29
33
Females. Total.
70
38
13
51
19
32
30
32
102
41
01
69
The Somerset County Lunatic Asylum has now been opened for the
reception of patients three years, and the committee speak, from expe-
rience, of its beneficial effects to that afflicted class of persons for whose
good the legislature ordered such buildings to be erected. It is grati-.
fying also to hear that its advantages are appreciated by the public.
Many persons from different parts of the county have visited it, and
their entries in the visitors' book show their satisfaction at the
comforts afforded to the patients, and the manner in which the establish-
ment is conducted.
" On the 31st December, 1849, the number of patients remaining in the
house was 286, since then 131 have been admitted; 117 of these have
been new cases, and 14 re-admissions; 64 discharged, and 34 have died;
there are now 319 remaining. Average number in the asylum during the
year, 294: 136 males,' and 158 females. One convalescent patient is out
with his friends, for one month, on trial, at the expiration of which time he
will be discharged, if his convalescence should continue."
It is observed, when speaking of the medical treatment of epilepsy, that
" Benefit has been derived from the use of a tincture of sumbul,
which has very much the odour of musk or castor, and has been lately
recommended in this disorder. One patient has lately been discharged,
who was long subject to most severe and frequent epileptic fits, and
had been for many months an inmate of the infirmary; after using this
medicine a short time, the fits diminished in frequency, and she had
but one very slight attack in the three months preceding her departure
from the asylum. At her own earnest solicitation and that of her
husband, she was allowed by the visitors to go home a month on trial:
that time has just now elapsed, and she has been discharged relieved.
TREATMENT OF EPILEPSY. 5G5
I do not think it likely that slie will continue well, as tlie disorder
seemed to be established and constitutional in her case; four out of
eight of her children died in convulsions, two of the four now living
are, like the mother, epileptic, and one of them, a daughter, was lately
so violent, that her father told me he thought it would have been found
requisite to have brought her as a patient to the asylum. The con-
nexion in this case between epilepsy in the mother, and convulsions in the
children, confirms what I have in former years observed, namely, a here-
ditary tendency between infantile convulsions, epilepsy, and insanity.*
A high medical authority, Van Sweiton, states, that persons who have
become insane at an early age, have been generally first epileptic. Esquirol
has come to a similar conclusion. Epilepsy is considered incurable, and
the treatment of it in a great measure empirical, unless, perhaps, when it is
symptomatic of disease of the circulating, digestive, or generative organs;
a great variety of remedies from all the kingdoms of nature have been
recommended, and many of them have long fallen into disuse. In some
instances aperients are found to alleviate the severity of the fits, and
attention to the diet is also of importance. It is a functional disorder of
the brain and spinal cord, and the symptoms, though so severe as to cause
death, and that suddenly, often leave no post-mortem change from what is
considered the ordinary healthy condition of those parts. No doubt
changes from the ordinary state are found frequently in the skull, in the
membranes of the brain and spinal cord, and in the structure of these
nervous centres themselves, in cases of epilepsy; but the same changes
are found in the bodies of those who had never been the subject of epi-
lepsy. The same is also the case with respect to insanity. The changes
which are ordinarily described as found in the brains of the insane, I have
again and again observed in the brains of persons who had never
been insane. Some of those changes, such as thickening and opacity of
the membranes with an increased quantity of fluid in the brain, I believe
to be a natural decay, which may be premature or the effects of old age,
when the brain becomes diminished in size. In epileptics there is some-
times found a partial absorption or diminution in the size of the brain, and
on the other hand it is sometimes found enlarged. The last male epileptic
who died in the asylum, was a young man aged twenty-three, affected from
childhood: previous to his disease a rapid succession of fits came on,
which, with some intermission, continued for three days; his respiration
was unusually laboured, indeed, almost suspended at times, with frothing
from both nose and mouth in large quantity. The greatest peculiarity in
the case was the very great size of the brain; it appeared to be almost
too large for the skull, and weighed 31b. 6oz. Another rapid case occurred
this year in a boy, aged fourteen, a congenital idiot; he was of a healthy
family, the fourth of eleven children; his mother had a fever, and was in
a baa state of health for six months preceding his birth. At the age of
twelve he became violent and dangerous to his younger brothers and
sisters, and was sent to the asylum. He was this year for the first time
attacked with epilepsy, and had only two fits at considerable intervals.
Previous to the attack which caused his death, on the one day he had
twelve severe fits, the following morning he fell into an insensible state,
and died about noon. The head was unusually large, the forehead large
and rather prominent; there was congestion of blood in the vessels of the
brain; this was above the average size of an adult's, and weighed upwards
of 31bs."
Dr. Boyd has drawn up his tabular statements with great care. We
* " Edinburgh Medical and Surgical Journal," No. 171, p. 452.
566 NORTHAMPTON LUNATIC ASYLUM.
wish it were in our power to extract a few of them. The result of the
post-mortem examinations are given minutely, and constitute an
important document-
In the Report of the Retreat at Bloomfield, near Dublin, we find the
following statement of the number of patients under treatment during
the year ended 31st of Third month last, 1851:?
Males. Females. Total.
In the house, 31st of Third month, 1850. ... 11 ... 12 ... 23
Admitted during the year  3 ... 8 ... 11
14 ... 20 ... 34
Males. Females. Total.
Discharged cured, or very much
improved 5 ... 3 ... 8
Removed by direction of the com-
? mittee, an old patient, previ-
ously in other asylums, incur-
able, and very troublesome to
all her fellow-patients ... 0 ... 1 ... .1.
_ __ 5 ... 4 ... 9
In the house, 31st of Third month, 1851 ...... 9 ... 16 ... 25
The Northampton Hospital for the insane materially differs from all
similar institutions throughout England. Receiving its inmates from
all parts of the kingdom, it practises no exclusion, its portals being
opened alike to the rich and the poor. Without any endowment, it is,
in the strictest sense of the word, a self-supporting institution: whilst
all are received, all contribute in varying proportions to their own
maintenance, and the sustentation of the fabric. Opened in the year
1838, it owes its origin to the pure-minded and voluntary efforts of
the inhabitants of the county of Northampton, who, following the
noble example of the second Earl Spencer, raised the present magni-
ficent structure. Uncontaminated by county rates, it nevertheless
receives, by the tacit consent of all parties, the paupers of the county,
on the fundamental principle that the inhabitants of the county having
been the largest contributors to the erection, had the greatest claim,
cceteris paribus, to consideration. Of the numbers so admitted on the
application of the county parishes, 150 may be considered to be the
average, the charge for the maintenance of each being 7s. Gd. a week,
with the average weekly addition of about 9d. for clothing; thirty-five
represents the number of out-county paupers, whilst about seventy-
five are private patients. It will thus be seen that Northamptonshire,
wisely anticipating the recent Acts of Parliament, has an institution
affording accommodation for about 260 persons?not only amply suffi-
cient for all the wants of the district, but extending the sphere of its
operations into distant provinces. Situated on high ground, within
half a mile of the town of Northampton, and surrounded by thirty-six
acres of its own freehold land, in the midst of picturesque scenery, it
WARNEFORD LUNATIC ASYLUM. '567
derives stability from the countenance of the principal nobility and
gentry of the county, who periodically visit and inspect it.
What the rate-payer has been compelled to do in other counties, the
efforts of individuals have voluntarily done in this. Of an institution
so happily conceived in its origin, and so widely diffusing its blessings,
let us express a hope that with its growing prosperity, some portion of
its funds may be hereafter set aside to help the well-educated and
friendless, who now, from exhaustion, are compelled to languish as
paupers in our county asylums amidst the degrading associations of
original poverty. As a class, there is none more pre-eminently entitled
to the sympathies of all good men than that of governesses. We
would specially recommend this class as worthy of every high-minded
consideration, to those who manage the pecuniary affairs of the North-
ampton Hospital for the Insane.
The following is an extract from the state of the institution of Saint
Thomas Hospital, near Exeter, for lunatics:?
State of the Patients, from the 1st July, 1801, (when the Hospital
was opened,) to the 25th March, 1851.
Admitted.
1497 Patients to tlie 25 th March, 1850.
18 Patients from the 25tb March, 1850, to 25th March, 1851.
1515 Discharged.
Recovered  775 \
Then on trial, since recovered 5 5
At the request of friends . . .
At the request of parishes . . .
Improper objects
Deceased
Incurable
Agreeably to the resolution of
the General Court of the 19th
of October, 1825 ....
Remain in the Hospital, 39, of
whom 24 are better, and 15
nearly as when admitted . .
Patients . . .
From
30th June, 1801,
to
25th March, 1850.
780
278
111
32
140
30
85
From
25th March, 1850,
to
25th March, 1851.
In all
to
25th March,
1851.
787
281
111
32
141
30
88
39
1515
The Warneford Asylum is altogether a voluntary institution; in no
part or period of the undertaking did it ever receive assistance from
any county purse; neither is it one of those asylums in which, by
virtue of certain provisions in the Act 48 G. III. c. 96, one part is
568 THE RETREAT AT YORK.
appropriated to county and parish purposes, and the rest reserved for
the charitable designs of the voluntary contributors. From its inde-
pendence in all these respects, it follows, that it is not in any sense a
county asylum. It is not a place of reception for pauper, or of impri-
sonment for criminal or felonious lunatics, of which descriptions there
are none within its walls.
Neither is this an asylum engrafted, like those at Manchester and
Leicester, upon an hospital or infirmary, and as such, built in close con-
tiguity to it. This, on the contrary, is a distinct and separate as well
as independent establishment. It stands on the rising ground, about a
mile and a half to the eastward of Oxford, and half a mile from the
London road on that side, and is supported partly by the reduced
weekly payments of the patients, and partly by subscriptions, donations,
and legacies, given or bequeathed by the benevolent, for the purpose of
enabling the institution to reduce or lower such weekly payments, in
aid of poor patients from those classes of society which are specially
described in a following page. And with respect to internal ministra-
tion and government, it is under the superintendence of its own physi-
cian, (a distinguished professor of the University of Oxford), and the
direction of its resident physician, its general care and control having
been placed by the rules of the society in a small committee of
management.
In this its character of a voluntary and self-supporting, integral, and
independent house, benevolently instituted for the care and treatment
of the insane, it has been recognised by the commissioners.
From a general meeting of the directors and friends of The Retreat,
held in York, the 24tli of Gth month, 1851, we arc glad to glean a
favourable report of this asylum. It also appears that
" The number of patients at present under care is 115; the average
number during the year has been 112*o, which is an increase of one upon
the average of last year. Fourteen of the patients are unconnected with
the Society of Friends. For patients of this class many applications
have been unsuccessfully made, owing to the apartments devoted to them
having been full during the whole of the year, from which cause only two
persons of this class have been admitted, one male and one female.
"Eleven persons, members of the Society of Friends, or closely con-
nected with it, have been admitted in the same time, making in all thirteen
admissions. Nine of these are persons who have been admitted for the
first time, and the remainder are re-admissions."
It is stated that
" Mechanical restraint has not been employed in a single instance
during the year as an aid in the treatment. It was used in one case as a
surgical appliance to a male patient with diseased feet, who persisted in
tearing off the dressings, but it was afterwards superseded by another
contrivance. The almost entire disuse of restraint during the twelve
months is not mentioned as reflecting credit on the management, but as a
simple fact."
SURREY LUNATIC ASYLUM. 509
From tlie last Report of tlie Surrey County Asylum we extract the
following statement of the medical statistics for the year 1849 :?
Males. Females. Total.
There were in tlie Asylum on 31st Dec. 1848. . 188 ... 21*2 ... 400
There have been admitted from that date to 31st
December, 1840   183 ... 258 .. 441
Total .... 371 ... 470 ... 841
Of this number there hare been discharged?
Males. Females. Total.
Recovered 39 ... 29 ... 68
Died  20 ... 35 ... 01
Removed uncured 2 ... 9 ... 11
07 ... 73 ... 140
Remaining iu the asylum 31st Dec. 1849 . . 304 ... 397 ... 701
" Of the patients admitted in 1849, some of them being cases of long
standing, 34 males and 23 females have been discharged recovered, and 11
males and 19 females have died.
" The number of patients under treatment in 1849 is considerably greater
than in any preceding year; having been?
In 1847   per cent.
1848    . , . ?
184 9
" This increase of the relative number of recoveries is in some measure
ascribed to the early removal of persons attacked With the disorder, from
the causes which have given rise to and tend to keep up their erroneous
ideas, as well as to medical treatment, directed to restore general health,
and judicious moral management, comprising useful occupations, and
innocent and instructing amusements, whereby the patient's attention is
withdrawn from erroneous or distressing subjects of thought."
It appears that the mucli-vaunted non-restraint system of treatment
does not find blind adherents in this asylum, as we should infer from
the following case, cited by the consulting physician of the establish-
ment, in his Report:?
" Nor can I, (Sir A. Morison,) although averse to any restraint what-
ever, when it can be avoided, altogether overlook the striking benefit that
in a few cases was the consequence of restraint, both personal and by
seclusion, employed for a short period, by which the train of morbid ideas
and the unlimited indulgence of morbid propensities appear to have been
interrupted, and reflection on surrounding objects induced in the patient's
mind, followed by a speedy recovery."
" * It would appear from the Reports of the four great public Establishments for the
reception of the Insane in the Metropolitan District, that the per centage of recoveries
1847
1848
1849
Bethlem
Hospital.
20M*
St. Luke's
Hospital.
25^
Hanwell
Asylum.
3 i7o3A
Springfield
Asylum.
o m
570 HAN WELL LUNATIC ASYLUM.
We have to thank Dr. Begley for a copy of the Sixth Report of the
Ilanwell County Asylum for 1851.
We are glad to perceive that the committee have passed a resolution
highly eulogistic of Dr. Hitchman, and expressing regret at his resigna-
tion of the office of one of the resident medical officers of the asylum.
The total number of patients in the asylum on the 1st of January,
were 1087?males, 483; females, 604.
We extract the following statement of the salaries given to some of
the principal officers connected with this institution:?
Per Annum.
Visiting physician ?315 0 0
Resident medical officer, males  200 0 0
1 Ditto females  200 0 0
1 Dispenser  70 0 0
1* Chaplain  250 0 0
1* Clerk to committee of visitors  100 0 0
1* Clerk of tlie asylum  300 0 0
2* Assistant-clerks, ?70 and ?G0   .130 0 0
1 Storekeeper  125 0 0
1 Assistant ditto  40 0 0
If Engineer   120 0 0
1* Schoolmaster  90 0 0
1 Matron  200 0 0
1 Assistant ditto  30 0 0
1 Housekeeper  GO 0 0
1 Superintendent of bazaar  25 0 0
1 Ditto of workroom    25 0 0
1 Ditto of laundry  25 0 0
19 ?2305 0 0
We glean the following interesting historical particulars of St. Luke's
Hospital for lunatics from the Report of the physician for the year 1850.
The original institution was on the north side of Upper Moorfields,
called Windmill-hill, where a mill formerly stood, facing what is now
called Worship-street. The estate was leasehold, held of the Corpora-
tion of London; and as the accommodations were not sufficiently
extensive to receive more than 110 patients, it was deemed most
advantageous to suffer the lease to expire, and to seek a larger ground-
plot on which a more commodious building might be erected.
The present institution originated in the benevolent designs of a
few gentlemen who saw the necessity of further provision for poor
lunatics; " for," as it is worded in the original circular, " there is no
disease to which human nature is subject so terrible in its appearances,
or so fatal in its consequences; those who are melancholy often do
violence to themselves,?and those who are raving to others, and too
often to their nearest relations and friends, the only persons who
cau be expected to take the trouble of these unhappy objects upon
them."
* Neither boarded nor lodged. + Lodged only.
ST. LUKE'S LUNATIC ASYLUM. 571
In the beginning of June, 1750, the above circular was subscribed
by- several gentlemen, who, on the 13th of the same month, met
together to consider the means of establishing a hospital for the said
purpose.
The subscribers having been considerably increased, met a third
time on the 12th of September, chose a committee, and empowered
them to take such steps as they should think necessary to forward this
good work. A fourth general meeting was summoned on the 10th of
October, when an account was opened with Messrs. Honeywood and
Fuller, and with Messrs. Drummond, to receive subscriptions for the
erection of the present building.
We are glad to chronicle the benevolent feelings of those who a
century ago laid the foundation of so noble a charity; and be it remem-
bered that these same feelings have wrought great changes in our times
in the condition and treatment of the poor lunatic. The hospital, no
doubt, was built according to the opinions, possibly the prejudices, of
those times. Tradition seems to have handed down to our ancestors a
monastery as the proper model for a lunatic asylum. The first that was
built was at Jerusalem, by the monks of the sixth century ; and the long
galleries and solitary rooms of Bethlem and St. Luke's seem to point to
the corridors and cells of the monastery as their original type; but,
however this may be, it is undoubtedly fortunate that our ancestors had
not a better model,?and it ill becomes those who possess the advan-
tages of modern improvements to speak lightly of the efforts of those
who were actuated by the same benevolent motives which have effected
so much good in ameliorating the sad condition of the insane.
The present building was commenced on the 30th of July, 1782; it
was erected by voluntary contributions at an expense of about 50,000?.
upon leasehold ground belonging to St. Bartholomew's Hospital; the
lease is held for a term of forty years, renewable every fourteen years on
payment of a fine of 200?., and -at the yearly rent of 200?.
It does not appear that boarders, or those deemed incurable, were
admitted into the hospital till 1754; at first only ten were admitted at
the rate of 5s. per week, and from that time till the year 1795, the
committee were authorized to admit 110 such patients for the same
sum.
The following is the average per centage of patients discharged cured
from St. Luke's Hospital:?
From 1821 to 1830   47 l per cent.
From 1831 to 1840   50^ ?
From 1841 to 1850  COf ?
When the hospital was first opened for the reception of patients, Dr.
Battie was its physician; in his time, and in that of Dr. Thomas Brooke,
his successor, six apothecaries supplied the medicines to the patients
572 ST. LUKE'S LUNATIC ASYLUM.
gratuitously. It would appear from reference to some of the old books
tliat the medical treatment consisted principally in anti-spasmodics and
purgatives; and the patients seem to have escaped the practice, at one
time prevalent in the treatment of lunacy, of being bled and purged
periodically every spring and fall. But a time arrived when the phy-
sician appointed to the hospital had no faith in medicine in the treat-
ment of insanity, but relied chiefly upon moral treatment, upon good
diet and exercise, and upon the occasional use of purgatives for effecting
a cure; and Ave find, by referring to our tables, that the average per
centage of recoveries during this period, i. e., from 3 791 to 1800, was
11| per cent, lower than between 1831 and 1840. This fact alone,
without reference to any other considerations, woidd have been sufficient
to have convinced us of the importance of attending to the medical
treatment of the patients confided to our care; and Ave are of opinion
that the moral treatment being the same, and other things being
assumed equal, the number of recoveries Avill advance pari passu with
the improvement in our knoAvledge of the pathology and medical treat-
ment of the disease.
In the Eeport before us, it is remarked that "Dr. Warburton Avas the
first physician in this country avIio prescribed morphia" in the treat1-
ment of insanity. It is also observed that " this medicine has been
considered by some as a specific in all cases of insanity." By Avhom ?
we Avould ask. Certainly, by no physician of experience or position.
In a certain class of mental affections, associated Avith a depressed con-
dition of the vital and nervous energies, and a sleeplessness at night
and restlessness by day, the persevering and continuous, not the occa-
sional, exhibition of morphia, has been found of essential benefit in the
cure of insanity. Its indiscriminate administration all Avould condemn.
We are acquainted Avith no physician Avho considers morphia " as a
specific in all cases of insanity." Dr. Seymour speaks highly of the
effects of this sedative in certain forms of mental disease, and Ave
could cite the particulars of a vast number of cases, many pronounced
to be lost and incurable, tohich have been restored to sanity by this mode
of treatment. Dr. Seymour protests against the indiscriminate and
exclusive use of morphia; and so do Ave; and therefore Ave think Avith
the physicians of St. Luke's Hospital, that " Avere Ave to be reduced to
the employment of one medicine only as a specific for all cases, Ave
should consider that Ave Avere going back to those times Avhen insanity
Avas supposed to be cured by hellebore, or to the dark ages, Avhen it Avas
treated by exorcism."
Restraint is never necessary in the treatment of the insane, is the
assertion of some. "What say Drs. Sutherland and Philp to this
dogma 1
" We should be deceiving- the peofession and the tublic if we
BRITISH LUNATIC ASYLUMS. 573
were to say that the result of our experience leads us to the belief that
restraint can be abolished with advantage to the patient in all cases, and
under all circumstances. But in saying this we distinctly repudiate the
notion of encouraging by our example any return to the cruel method of
treatment which was formerly practised in this and in other countries; and
we assert that we feel no sympathy with those who employ restraint merely
for the purpose of saving trouble to themselves and attendants. There is
no general rule without its exceptions, and we conscientiously think that
there are some exceptions to the total abolition of coercion, not only in private
practice where there are no means and appliances at hand for the treatment
of the paroxysm, but even in asylums also. The exceptions to our general
rule of the non-employment of coercion in the hospital amount according
to the daily report to two in 100."
We subjoin a list of those gentlemen who have held the office o
physician to the hospital from its foundation, together with the date
on which they were respectively appointed:?
"William Battie, M.D., October 31st, 1750.
Thomas Brooke, M.D., April 19th, 1764.
Samuel Foart Simmons, M.D., November 8th, 1781.
Alexander Robert Sutherland, M.D., March 16th, 1811.
John Warburton, M.D., May 19th, 1829.
Alexander John Sutherland, M.D., March 25th, 1841.
Erancis Richard Philp, M.D., June 22nd, 1842.
We append a statement of the number of patients admitted and dis-
charged from 1st January to 31st December, 1850.
Males. Females. Total.
In tlie hospital on 1st January, 1850   3D ... 01 ... 100
Admitted during the year 72 .... 107 .... 179
Males. Fem. 111 *68
Discharged, unfit 10 .... 0
? by desire of friends ... 1 ... 2
Remaining in the hospital 35 ... 53
? ? 4G ... 61 ... 107
Treatment completed  05 ... 107 .... 172
Males. Females.
Cured . . 44 equal to 07*09 per cent.
Uncured . 17 ? 20*15 ?
Died ... 4 ? 0*15 ?
Cured . .. 09 equal to 04*48 per cent.
Uncured. . 29 ? 27*10 ?
Died ... 9 ? 8 41 ,,
Males and Females together.
Cured 1J3 equal to 05*09 percent.
Uncured .... 40 ? 20*74 ?
Died  13 ,, 7*55 ,,
The thirty-second Report of the Staffordshire General Lunatic
Asylum for 1850, gives us the following general statement of patients
admitted, discharged, and now on the books, from the opening of the
asylum, October 1st, 1818, to December 31st, 1850:?
Total number of admissions 3481
Discharged recovered    1520 \
Ditto relieved  475 I ggoo
Removed, as harmless or incurable, or by desire of friends . . 493 (
Died  732 )
Remaining under cure 30 )
Ditto incurable 231 J
NO. XVI. P P
574 MEDICAL EVIDENCE IN CASES OF INSANITY.
We had marked many more passages for extraction from the body of
Keports before us, but we were warned by the printer's devil that Ave
had already exceeded our limits, and were reluctantly compelled to
throw aside our pen. We must reserve some general remarks for
another occasion.

				

